# Is it time to stop sweeping data cleaning under the carpet? A novel algorithm for outlier management in growth data

**DOI:** 10.1371/journal.pone.0228154

**Published:** 2020-01-24

**Authors:** Charlotte S. C. Woolley, Ian G. Handel, B. Mark Bronsvoort, Jeffrey J. Schoenebeck, Dylan N. Clements

**Affiliations:** 1 The Roslin Institute, The University of Edinburgh, Easter Bush Campus, Midlothian, Edinburgh, United Kingdom; 2 The Royal (Dick) School of Veterinary Studies, The University of Edinburgh, Easter Bush Campus, Midlothian, Edinburgh, United Kingdom; Liverpool John Moores University, UNITED KINGDOM

## Abstract

All data are prone to error and require data cleaning prior to analysis. An important example is longitudinal growth data, for which there are no universally agreed standard methods for identifying and removing implausible values and many existing methods have limitations that restrict their usage across different domains. A decision-making algorithm that modified or deleted growth measurements based on a combination of pre-defined cut-offs and logic rules was designed. Five data cleaning methods for growth were tested with and without the addition of the algorithm and applied to five different longitudinal growth datasets: four uncleaned canine weight or height datasets and one pre-cleaned human weight dataset with randomly simulated errors. Prior to the addition of the algorithm, data cleaning based on non-linear mixed effects models was the most effective in all datasets and had on average a minimum of 26.00% higher sensitivity and 0.12% higher specificity than other methods. Data cleaning methods using the algorithm had improved data preservation and were capable of correcting simulated errors according to the gold standard; returning a value to its original state prior to error simulation. The algorithm improved the performance of all data cleaning methods and increased the average sensitivity and specificity of the non-linear mixed effects model method by 7.68% and 0.42% respectively. Using non-linear mixed effects models combined with the algorithm to clean data allows individual growth trajectories to vary from the population by using repeated longitudinal measurements, identifies consecutive errors or those within the first data entry, avoids the requirement for a minimum number of data entries, preserves data where possible by correcting errors rather than deleting them and removes duplications intelligently. This algorithm is broadly applicable to data cleaning anthropometric data in different mammalian species and could be adapted for use in a range of other domains.

## Introduction

Since the turn of the millennium, there has been an explosion in the amount of data available to the public, industry and academia. However, it has been acknowledged for over 50 years that large, computer-based datasets contain errors due to transcription, coding and misunderstandings [1]. Ignoring extreme errors has substantial adverse effects on data summaries [2], statistical tests [3] and may invalidate results [4]. In studies identifying risk, errors increase the variance of the covariate and lead to regression dilution [5]. To remove errors, data cleaning is required: defined as the “process of detecting, diagnosing, and editing faulty data” [6]. Ideally, data cleaning methods should prioritise data repair over data removal [7] and use computer programs to improve reproducibility [8].

It is difficult to distinguish errors from genuine anomalies in certain types of data, such as height and weight records, because biological data is heterogenous and may contain unusual but plausible values. These datasets are variable in terms of how accurate they are, with authors estimating error rates to be anything from 0.03% to 4.5% [9–13]. Since the first computational cleaning method for longitudinal growth [14] there have been enormous technological advancements, yet there remains no standardised data cleaning method. A review of 42 studies including growth parameters reported that 41% did not describe data cleaning and a further 26% described methods that were not reproducible. The methods used by the remaining percentage were very different from each other and when tested on the same dataset detected between 0.04% and 1.68% errors; a 42 fold difference [15].

Many researchers have used externally defined limits to identify implausible values, such as outlier cut-offs based on arbitrary values, guides defined by the WHO and growth charts published by the CDC [9,16–21]. However, cut-offs like these have poor specificity and can underestimate population change such as the increasing prevalence of obesity [22]. In other domains, such as veterinary epidemiology, externally validated information is rarely available and is usually species or breed specific. Other authors have reported using internally defined cut-offs that rely on the average to remove outliers. Simple examples include removing all values with a z-score of less than or more than three [23] or five [24] or more than 1.5 box lengths away from the 25^th^ or 75^th^ percentile using Tukey’s method [25]. This approach has been enhanced by adding age bins [26] or algorithms [27] to account for age-related shifts in weight. However, these methods are specific to the studies they were designed for and rely on population averages, which are distorted by extreme values and do not account for individual variation.

Longitudinal data cleaning methods (those that consider an individual’s other data points) are becoming more common but are widely variable. SITAR (Superimposition by Translation And Rotation) [28] and the ‘Outliergram’ [29] are visualisation methods that allow individual trajectories to be viewed but are specific to each dataset they are applied to and require subjective judgements to be made, which can be time consuming when applied to large datasets. Algorithms that examine the change between two measurements are simple to apply in comparison with many longitudinal methods but are limited by poor specificity and are not cable of identifying consecutive errors [30]. Daymont and colleagues designed an automated data cleaning technique based on exponentially weighted moving average standard deviation scores combined with a decision-making algorithm to identify implausible growth data. The method deals with erroneous duplications, aims to correct errors rather than exclude them and has been validated by simulating errors and obtaining physicians reviews [12]. However, despite high sensitivity (97%), the method could not detect errors in the first or last measurements or in highly erroneous individuals and the specificity of the method was relatively low (90%). A study that compared this method with a regression-based weight change model [31] and another method based on standard deviation scores [32] demonstrated that all methods had good specificity (>98%) and poor sensitivity (<19%) [33]. Daymont and colleagues’ method performed the worst, indicating it is not as effective on other datasets as the one it was originally designed for. Other longitudinal methods have had similar limitations. Yang and Hutcheon [11] published a conditional growth percentile method that predicts a weight percentile at time *t* based on the individual’s weight percentile at time *t-1* but cannot be used on an individual’s first measurement and does not identify consecutive errors. Shi, Korsiak, & Roth [34] used a jack-knife residual method, which had a higher sensitivity and specificity when compared to Yang and Hutcheon’s method but could only be used on individuals with at least four measurements. Linear mixed-effects models have been used to identify erroneous weight measurements in human adults [35] and have been adapted for use in growth data by combining sex-stratified, mixed effects, linear spline regression models with externally defined z-score cut-offs [13]. However, the method is unvalidated so it is difficult to assess its effectiveness and it has many of the limitations of other approaches; it does not address duplications, it removes errors rather than corrects them and it is specific to the study population.

In summary, despite numerous attempts by many researchers to produce a data cleaning method capable of detecting erroneous growth values, there is no ‘gold standard’ and many methods have limited applicability. Furthermore, few data cleaning methods [12, 32] for growth address duplication in addition to error, which is well-documented to be an issue in electronic databases [36–38]. Based on these observations, our primary aim was to develop an adaptable, computer-based data cleaning algorithm that could be applied to a variety of growth datasets. We required the algorithm to use pre-applied cut-offs to influence decision making, to include de-duplication, to prioritise data repair over data removal, to be effective on consecutive errors and to operate despite the number of data entries per individual. Our secondary aim was to simulate different types of artificial errors into a pre-cleaned dataset and compare the data preservation, sensitivity, specificity and convergence of five commonly used data cleaning approaches with and without the algorithm.

In this paper, we describe the five datasets we used to test our algorithm and report how we identified and subsequently simulated errors in this data. We demonstrate that our novel data cleaning algorithm improves the performance of five commonly used methods for identifying implausible values in growth data. Finally, we apply the method with the highest performance to all five datasets.

## Materials and methods

We follow the STROBE [39] and RECORD [40] Statements in reporting this study. All data analysis was carried out using R statistical software. An example of the code, including the specific packages and functions used for this study, is available at https://github.com/CharlotteWoolley/growth_cleanR.

### Data sources

**Fig 1:**
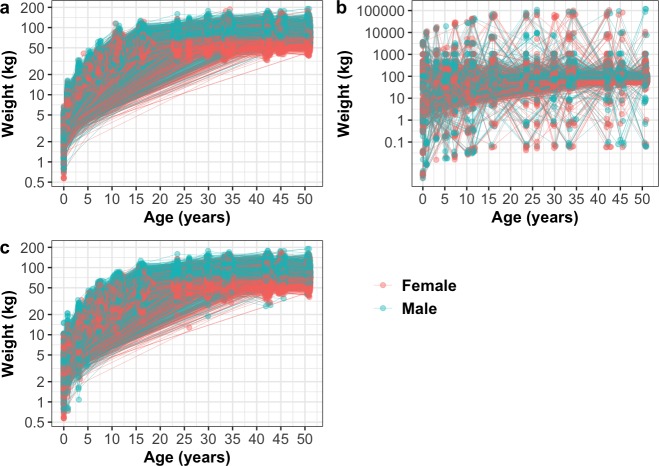
**Weights of humans by age in CLOSER data without simulated duplications and errors (a), with simulated duplications and 1% errors prior to data cleaning (b) and with simulated duplications and 1% errors after data cleaning with the NLME-A method (c).** Duplications were simulated by randomly selecting 2.5% of the data and duplicating it once, followed by randomly selecting a further 2.5% of the data and duplicating it twice. Simulated errors were made up of 50% random errors and 50% fixed errors. Random errors were simulated between the values of 0.0001 and 500. Fixed errors comprised of manipulating measurements by multiplying and dividing by 10, 100 and 1000, adding 100 and 1000, converting to the metric and imperial units and transposing the number.

A brief description of the study design, data collection, cohort details and data accessibility of the five datasets used are given in Table 1. Dogslife was approved by the University of Edinburgh Veterinary Ethical Review Committee (Ref: 7.5.09) and Human Ethical Review Committee (Ref: HERC_161_17). Further detailed information about Dogslife data collection is given in S1 File. The Small Animal Veterinary Surveillance Network (SAVSNET), Banfield and Cohort and Longitudinal Studies Enhancement Resources (CLOSER) data were obtained from third party sources and the information for their relevant ethical approvals and data collection methods can be obtained from the relevant citations given in Table 1. CLOSER data is shown in Fig 1 and all other data are shown in S1 to S4 Figs.

**Table 1:** Description of the study design, data collection and processing, cohort details and data accessibility for longitudinal height or weight measurements in Dogslife, SAVSNET, Banfield and CLOSER datasets.

Details	Dogslife		SAVSNET	Banfield	CLOSER
Study design	A longitudinal, online study of the morphology, lifestyle and health of pedigree UK Kennel Club registered Labrador Retrievers in the UK [41]		A structured health surveillance program for UK companion animals through the collection of laboratory and veterinary clinical records [42]	A large-scale network of more than 1000 veterinary hospitals based primarily in the US that store electronic records from veterinary consultations [43]	A consortium of longitudinal studies based in the UK. Data used was harmonised from the 1958 National Child Development Study, the 1970 British Cohort Study and the Millennium Cohort Study [21, 44–49]
Data type	Owner-reported digital questionnaires		Digital records of veterinary consultations from 387 practices	Digital records of veterinary consultations from 652 hospitals	A combination of self-reports and data collected by health and scientific professionals
Species	Dog		Dog	Dog	Human
Breed classification	UK Kennel Club registered Labrador Retrievers		UK self-classified Labrador Retrievers	US self-classified Labrador Retrievers	*_ _ _*
Data collection period	July 2010 to June 2017		April 2014 and September 2017	October 1994 to March 2013	Various [30–36]
Data condition	Raw/uncleaned		Raw/uncleaned	Raw/uncleaned	Pre-processed/cleaned
Measurement type	Weight	Height	Weight	Weight	Weight
Age of cohort (Mean ± SD)	1.65 ± 1.55, (0.01–7.41)	0.71 ± 0.53, (0.01–5.26)	1.70 ± 1.88, (0.04–13.99)	0.70 ± 0.51, (0.00–2.00)	16.63 ± 15.80, (0.00–51.17)
Data accessibility	Available to download: https://doi.org/10.7488/ds/2569 [50]		Available on request: https://www.liverpool.ac.uk/savsnet/using-savsnet-data-for-research/	Available on request: https://doi.org/10.1371/journal.pone.0182064 [26]	Available to download: http://doi.org/10.5255/UKDA-SN-8207-1 [44]

The age of the cohort describes the mean plus or minus the standard deviation followed by the range of the age in years of individuals in the study.

### Error and duplication identification

All four of the datasets that were not pre-cleaned contained evidence of erroneous measurements and duplications. Suspected errors were identified by manually and visually examining the datasets for biologically implausible values, patterns (for example, where the incorrect unit had been used) and common typing discrepancies. Duplicate entries were identified as measurements that shared an individual’s identity and were entered on the same date. Removing duplications by keeping only the most recent data entry in a set of duplicates had an impact on the mean and standard deviation of the growth measurements in all datasets but was not sufficient to clean the data alone. This preliminary examination of the data created the basis for the design of our data cleaning algorithm. Table 2 describes the effect of removing duplications on the size, mean and standard deviation of the datasets.

**Table 2:** Description of the data entries, individuals, data entries per individual, mean and standard deviation of the longitudinal height or weight measurements in Dogslife, SAVSNET, Banfield and CLOSER data with and without simulated duplications and 1% errors before and after removal of duplicated measurement records.

Details	Dogslife		SAVSNET	Banfield	CLOSER	
	Weight	Height			Original pre-cleaned data	With simulated duplications and errors
*Before duplication removal*						
Data entries	43 421	28 012	49 893	17 447	236 564	255188
Individuals	5622	5521	5195	1974	42 803	42 803
Data entries per individual	17.52 ± 12.35, 1–76	9.01 ± 5.81, 1–59	13.56 ± 9.82, 1–74	10.37 ± 4.38, 5–32	6.13 ± 1.70, 1–9	6.75 ± 2.14, 1–19
Mean ± SD	25.71 ± 69.77	47.90 ± 12.50	23.72 ± 18.26	21.32 ± 10.65	41.03 ± 30.20	63.61 ± 1135.70
*After duplication removal*						
Data entries	37 482	23 498	44 362	17 313	*_ _ _*	236 564
Individuals	5622	5521	5195	1974	*_ _ _*	42 803
Data entries per individual	14.79 ± 10.12, (1–58)	7.00 ± 3.74, (1–27)	10.55 ± 5.13, (1–51)	10.28 ± 4.34, (3–32)	*_ _ _*	6.13 ± 1.70, (1–9)
Mean ± SD	25.36 ± 52.38	48.36 ± 11.91	23.70 ± 19.01	21.31 ± 10.65	*_ _ _*	65.42 ± 1179.51

Duplications were simulated by randomly selecting 2.5% of the data and duplicating it once, followed by randomly selecting a further 2.5% of the data and duplicating it twice. Simulated errors were made up of 50% random errors and 50% fixed errors. Random errors were simulated between the values of 0.0001 and 500. Fixed errors comprised of manipulating measurements by multiplying and dividing by 10, 100 and 1000, adding 100 and 1000, converting to the metric and imperial units and transposing the number. Data entries per individual describes the mean plus or minus the standard deviation followed by the range of the number of data entries inputted by each individual in the study. The mean ± SD describes the mean plus or minus the standard deviation of the growth measurements

### Error and duplication simulation in CLOSER data

CLOSER data was pre-processed and cleaned by the CLOSER authors prior to our receival of the data. In brief, subsets of the National Child Development Study, the 1970 British Cohort Study and the Millennium Cohort Study were selected based on bias-minimising criteria. They were merged and cleaned in Stata statistical software by replacing missing data where possible, attempting to correct for previously over-cleaned measurements and removing any data that were regarded as unaccountable or biologically implausible. Weight and height measurements were deemed as biologically implausible by using a combination of cut-offs (e.g. over 250kg) and scatter plot visualisation [21]. After we received the data, it contained no outliers or duplications upon examination.

To accurately simulate an unclean dataset so that we could test the sensitivity and specificity of various data cleaning methods with and without our algorithm, inaccuracies and duplications were randomly introduced to the CLOSER data. For all simulations, 2.5% of the data was randomly selected, duplicated once and added back to the data. A further 2.5% of this data was again randomly selected, duplicated twice and added back to the data. Twelve types of error were simulated by multiplying and dividing by 10, 100 and 1000, adding 100 and 1000, converting to the metric and imperial units, transposing the number (reversing the order of two digits) or selecting a random number between 0.0001 and 500. Errors were simulated for 0%, 0.1%, 0.2%, 0.5%, 1%, 2%, 5%, 10%, 20% and 50% of the data, where random number errors comprised between 0% and 100% (in 10% intervals) of the total errors and other errors made up the remaining percentage in equal proportions. This addition of 0% error rates allowed random and fixed errors to be simulated separately and in combination with each other. Sensitivity was calculated as the percentage of simulated (true-positive) measurement errors that were correctly identified and specificity was calculated as the percentage of non-simulated (true-negative) measurements that were correctly identified. We report CLOSER weights with simulated duplications and 1% simulated errors because we estimated that this was a realistic error rate for an unclean dataset based on previous research. We also report the average values across all different error simulations to demonstrate the applicability of methods to datasets with very low or high error rates. CLOSER weights prior to error simulation, with simulated duplications and 1% simulated errors (50% random and 50% fixed) and post-cleaning are shown in Fig 1.

### Data cleaning algorithm

**Fig 2:**
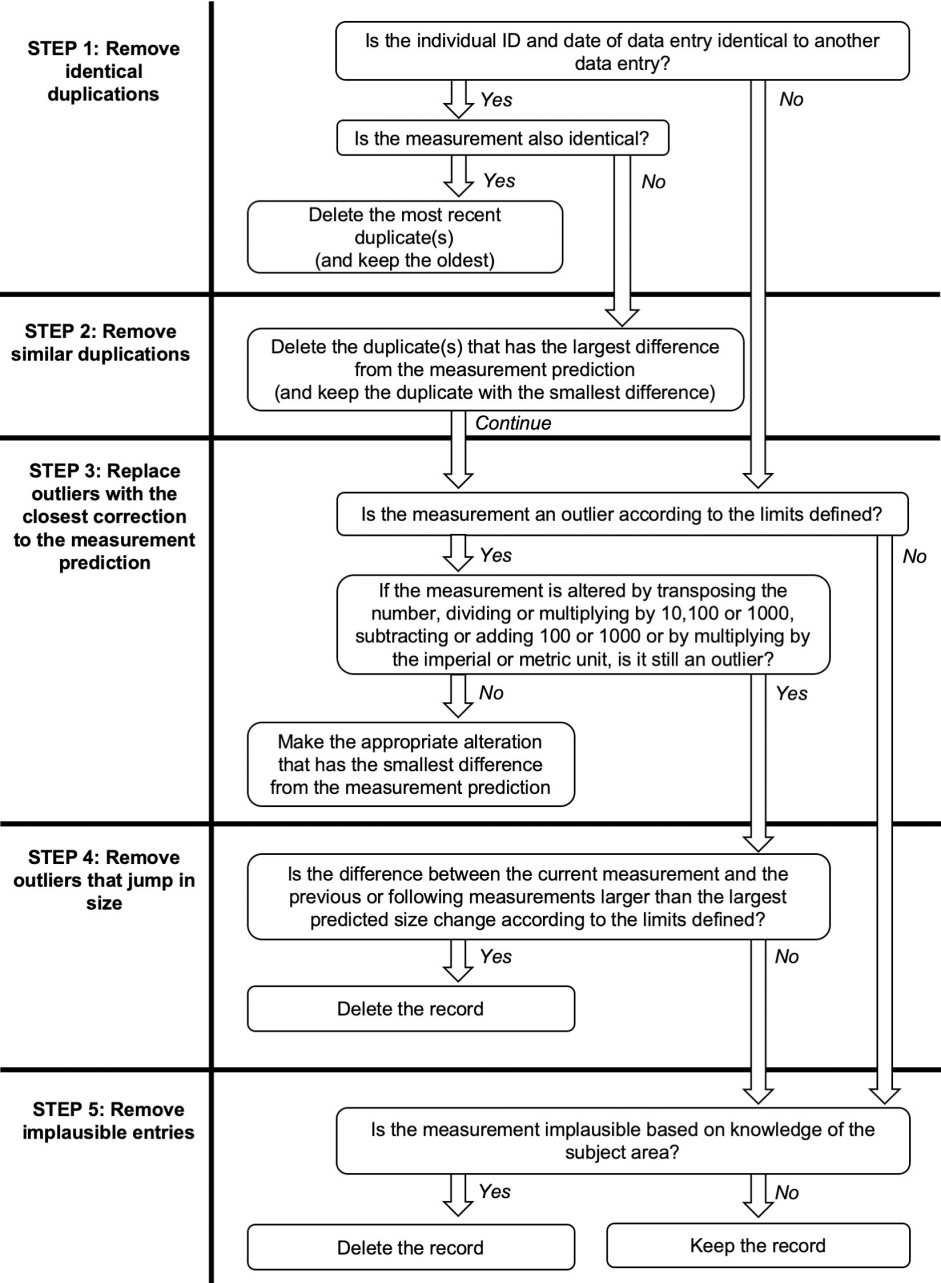
A five-step data cleaning algorithm for growth data that uses pre-defined measurement predictions and prediction limits to identify which measurements are likely to be erroneous and to make appropriate corrections and deletions.

A five-step data cleaning algorithm (see Fig 2) was designed to improve the performance of five standard data cleaning methods (see sections below), to correct, delete or retain measurements that were suspected to be erroneous and to preserve data where possible.

There were several definitions that had to be made based on the chosen data cleaning method prior to the application of the algorithm. Throughout, the algorithm required outliers to be defined by lower and upper measurement prediction limits. For steps 2 and 3, exact measurement predictions needed to be defined so that logical decisions using the most likely values could be made. In step 3, numbers were not transposed when the difference between the original and transposed numbers was 9 because we considered this value to be a biologically plausible deviation from the prediction limits in these datasets. For step 4, the largest predicted size change between two measurements was defined as the difference between the lower measurement prediction limit of the first data point and the upper measurement prediction limit of the second data point. For step 5, implausible measurements based on knowledge of the subject area needed to be defined. For dog weights, implausible measurements were considered as less than 0.5 kg and more than 90 kg and for dog heights, less than 3 cm and over 90 cm. For human weights, the upper limit was 250kg for all ages and two lower limits were set to account for premature births: less than 0.5 kg under the age of five years old and less than 10 kg for ages five years old and older.

### Data cleaning methods with and without the addition of our algorithm

#### General cut-off and general cut-off with algorithm

Outliers were defined in the same manner as for implausible measurements in step 5 of our data cleaning algorithm. In the general cut off (GCO) method, duplicates were removed by keeping only the most recent data entry in a set of duplicates and outliers were deleted. In the general cut off with algorithm (GCO-A) method, the measurement prediction was set as the average measurement defined by the American Kennel Club Labrador Retriever weight and height breed standards [51] and the Office for National Statistics UK average weight statistics [52] and the algorithm was applied to the data to make appropriate modifications and deletions in a step-by-step manner.

#### Standard z-score cut-off and standard z-score cut-off with algorithm

Outliers were defined as those with a z-score with an absolute value of greater than 3; otherwise described as values that were more than three standard deviations away from population mean. In the standard z-score cut-off (SZCO) method, duplicates were removed by keeping only the most recent data entry in a set of duplicates and outliers were deleted. In the standard z-score cut-off with algorithm (SZCO-A) method, the measurement prediction was set as the mean population measurement for that dataset and the algorithm was applied to the data to make appropriate modifications and deletions in a step-by-step manner.

#### Temporal z-score cut-off and temporal z-score cut-off with algorithm

Data were divided into age category bins from the minimum to the maximum age in the dataset. Age category bins were 30 days for dogs and 365 days for humans unless there were less than 100 data entries in that category, in which case the time period was increased by the appropriate age category bin until there were at least 100 data entries in each category. Outliers were defined as those with a z-score with an absolute value of greater than 3 within each age category. In the temporal z-score (TZCO) method, duplicates were removed by keeping only the most recent data entry in a set of duplicates and outliers were deleted. In the temporal z-score with algorithm (TZCO-A) method, the measurement prediction was set as the mean population measurement for that dataset and the algorithm was applied to the data to make appropriate modifications and deletions in a step-by-step manner.

#### Non-linear regression model cut-off and non-linear regression model cut-off with algorithm

We applied non-linear modified Gompertz regression models and a non-linear asymptotic regression model to the uncleaned weight and height data to provide measurement predictions. For further details of model setup and fitting, please refer to S2 File, S1 Table and S2 Table. Outliers were defined as measurements outside of the population predicted value plus or minus four times the standard deviation because values that exceed this boundary are considered ‘far outliers’ according to statistical convention [53]. In the non-linear regression cut-off (NLR) method, duplicates were removed by keeping only the most recent data entry in a set of duplicates and outliers were deleted. In the non-linear regression cut-off with algorithm (NLR-A) method, the algorithm was applied to the data to make appropriate modifications and deletions in a step-by-step manner.

#### Non-linear mixed effects model cut-off and non-linear mixed effects model cut-off with algorithm

We applied non-linear modified Gompertz mixed effects models and a non-linear asymptotic mixed effects model to the uncleaned weight and height data to provide measurement predictions. For further details of model setup and fitting, please refer to S2 File, S1 Table and S2 Table. The data was divided into age category bins as described in the TZCO method, data was simulated for each ID and sex at the mean age of each age category and measurement predictions were calculated for each simulation. The variation due to random effects was estimated as four times the standard deviation of these measurement predictions [53]. The residual variation was estimated as four times the ‘smoothed’ (using local regression) standard deviations of the residuals at each age category. Individual prediction intervals were estimated as the measurement prediction including random effects plus or minus the estimated residual variation. Where individual predictions were not possible, population prediction intervals were estimated as the measurement prediction for the population plus or minus the estimated variation of random effects in addition to the estimated residual variation. Outliers were defined as measurements that were outside of the individual prediction intervals when available and population prediction intervals when not available. In the non-linear mixed effects model cut-off (NLME) method, duplicates were removed by keeping only the most recent data entry in a set of duplicates and outliers were deleted. In the non-linear mixed effects model cut-off with algorithm (NLME-A) method, the algorithm was applied to the data to make appropriate modifications and deletions in a step-by-step manner.

## Results

### Comparison of the effect of different data cleaning methods on uncleaned datasets

Table 3 compares the effect of the five data cleaning approaches with and without our algorithm on the mean, standard deviation and preservation of data in longitudinal growth measurements from Dogslife, SAVSNET and Banfield. Most data cleaning methods resulted in data that had different means and standard deviations than the uncleaned data. The NLME method reduced the variation the most in Dogslife height and weight data, while the TZCO-A method reduced the variation the most in SAVSNET and BANFIELD weight data. The GCO-A method resulted in the highest or joint highest data preservation out of all methods in all datasets. Dogslife heights had the lowest percentage of post-cleaning data preservation (80.39% to 83.61%) whilst Banfield weights had the highest (98.74% to 99.23%).

**Table 3:** The mean, standard deviation and preservation of data (PD) of five data cleaning approaches with and without an algorithm (A) compared to uncleaned longitudinal growth measurements in Dogslife, SAVSNET and Banfield data.

Method	Dogslife				SAVSNET		Banfield	
	Weight		Height					
	Mean ± SD	PD (%)	Mean ± SD	PD (%)	Mean ± SD	PD (%)	Mean ± SD	PD (%)
Uncleaned	25.71 ± 69.77	100.00	47.90 ± 12.50	100.00	23.72 ± 18.26	100.00	21.32 ± 10.65	100.00
GCO	24.52 ± 8.80	86.20	48.48 ± 11.20	83.33	23.57 ± 9.93	88.90	21.31 ± 10.62	99.20
GCO-A	24.53 ± 8.82	86.33	48.46 ± 11.20	83.61	23.58 ± 9.92	88.91	21.31 ± 10.62	99.23
SZCO	24.57 ± 9.21	86.26	48.56 ± 10.92	83.00	23.57 ± 9.93	88.90	21.28 ± 10.59	99.15
SZCO-A	24.52 ± 8.81	86.26	48.39 ± 10.92	83.54	23.57 ± 9.92	88.91	21.28 ± 10.58	99.21
TZCO	24.46 ± 8.71	85.92	48.85 ± 10.50	81.91	23.54 ± 9.86	88.41	21.29 ± 10.58	98.80
TZCO-A	24.45 ± 8.68	86.30	48.69 ± 10.54	83.54	23.52 ± 9.84	88.91	21.29 ± 10.57	99.21
NLR	24.47 ± 8.69	86.11	48.99 ± 10.41	81.45	23.57 ± 9.93	88.90	21.30 ± 10.62	99.21
NLR-A	24.46 ± 8.70	86.32	48.96 ± 10.39	83.57	23.57 ± 9.92	88.91	21.30 ± 10.62	99.21
NLME	24.46 ± 8.63	85.49	49.22 ± 10.05	80.39	23.58 ± 9.91	88.51	21.31 ± 10.61	98.74
NLME-A	24.45 ± 8.66	86.10	49.25 ± 10.10	83.26	23.57 ± 9.91	88.77	21.30 ± 10.61	99.08

The mean ± SD describes the mean plus or minus the standard deviation of the growth measurements. The preservation of data (PD) describes the percentage of the original data that was preserved.

#### Comparison of the effect of different data cleaning methods on CLOSER data with simulated duplications and 1% simulated errors

Table 4 compares the effect of the five data cleaning approaches with and without our algorithm on the mean, standard deviation, preservation of data and the sensitivity and specificity of outlier detection in longitudinal growth measurements from CLOSER data with simulated duplications and 1% simulated errors. All data cleaning methods resulted in different means and standard deviations than the data with simulated duplications and 1% simulated errors. The NLME-A method resulted in a mean and standard deviation closer to the original data without simulated errors and duplications (0.07kg less than the original mean and 0.03kg less than the original standard deviation) than any other method. The GCO-A method resulted in the highest data preservation, the NLME-A method had the highest sensitivity and the NLR-A method had the highest specificity.

**Table 4:** The mean, standard deviation, preservation of data (PD), sensitivity and specificity of five data cleaning approaches with and without an algorithm (A) compared to uncleaned longitudinal growth measurements in CLOSER data with and without simulated duplications and 1% errors.

Method	Mean ± SD	PD (%)	Sensitivity (%)	Specificity (%)
Pre-cleaned without simulations	41.03 ± 30.20	_ _ _	_ _ _	_ _ _
Uncleaned with simulations	63.61 ± 1135.70	100.00	0.00	100.00
GCO	41.34 ± 30.86	92.15	56.38	99.96
GCO-A	41.43 ± 30.82	92.70	59.85	99.99
SZCO	43.39 ± 57.54	92.64	6.10	99.96
SZCO-A	41.32 ± 30.79	92.27	60.05	99.99
TZCO	42.61 ± 47.84	92.56	14.71	99.95
TZCO-A	41.28 ± 30.77	92.33	61.52	99.99
NLR	41.09 ± 30.39	92.18	53.51	99.95
NLR-A	41.10 ± 30.35	92.57	71.93	100.00
NLME	40.94 ± 30.16	91.64	86.00	99.75
NLME-A	40.96 ± 30.17	92.45	90.55	99.85

Duplications were simulated by randomly selecting 2.5% of the data and duplicating it once, followed by randomly selecting a further 2.5% of the data and duplicating it twice. Simulated errors were made up of 50% random errors and 50% fixed errors. Random errors were simulated between the values of 0.0001 and 500. Fixed errors comprised of manipulating measurements by multiplying and dividing by 10, 100 and 1000, adding 100 and 1000, converting to the metric and imperial units and transposing the number. The mean ± SD describes the mean plus or minus the standard deviation of the growth measurements. The preservation of data (PD) describes the percentage of the original data that was preserved. Sensitivity was calculated as the mean percentage of simulated (true-positive) measurement errors that were correctly identified. Specificity was calculated as the mean percentage of non-simulated (true-negative) measurements that were correctly identified.

Gold standard (GS) corrections during data cleaning can be defined as modifying an error according to the complementary method to which it was introduced. For example, the GS correction for an error that had been induced by multiplying a value by 1000 would be to divide the error by 1000 and for an induced random error it would be any modification to that measurement. Table 5 below reports the percentage of GS corrections made by the algorithm-based methods for the different error types in the CLOSER data with simulated duplications and 1% simulated errors. Non-algorithm-based methods are not reported as they are not capable of making corrections. The NLME-A method made more or equivalent GS corrections than all other methods in all error types except in random errors, and divide by 100 errors, where it made 5.88% and 2.59% fewer GS corrections respectively than the NLR-A method. The mean percentage of GS corrections across all error types was highest in the NLME-A method.

**Table 5:** The percentage of gold standard corrections of errors induced into CLOSER data with simulated duplications and 1% errors using the algorithmic data cleaning methods.

Induced error type	GS error correction	GCO-A	SZCO-A	TZCO-A	NLR-A	NLME-A
Random	Any	45.69	0	7.52	59.80	53.92
Transpose	Transpose	0	0	1.37	0	24.66
/10	x10	33.91	0	5.22	55.65	84.35
/100	x100	59.48	38.79	61.21	87.07	84.48
/1000	x1000	65.52	65.52	90.52	90.52	90.52
x10	/10	33.62	0	1.72	0	47.41
x100	/100	28.45	0	0	0	44.83
x1000	/1000	35.34	0	0	0	47.41
+100	-100	0	0	1.72	9.48	18.97
+1000	-1000	41.38	0	23.28	83.62	83.62
Metric	Imperial	0.86	0	0	0	22.41
Imperial	Metric	0	0	0	8.62	42.24
Average across all errors		28.69	8.69	16.05	32.90	53.74

Duplications were simulated by randomly selecting 2.5% of the data and duplicating it once, followed by randomly selecting a further 2.5% of the data and duplicating it twice. Simulated errors were made up of 50% random errors and 50% fixed errors. Random errors were simulated between the values of 0.0001 and 500. Fixed errors comprised of manipulating measurements by multiplying and dividing by 10, 100 and 1000, adding 100 and 1000, converting to the metric and imperial units and transposing the number. Gold standard (GS) corrections are defined as modifying a simulated error according to the complementary method to which it was introduced

#### Comparison of the mean effect across different rates and types of simulated errors and duplications of different data cleaning methods on CLOSER data

**Fig 3:**
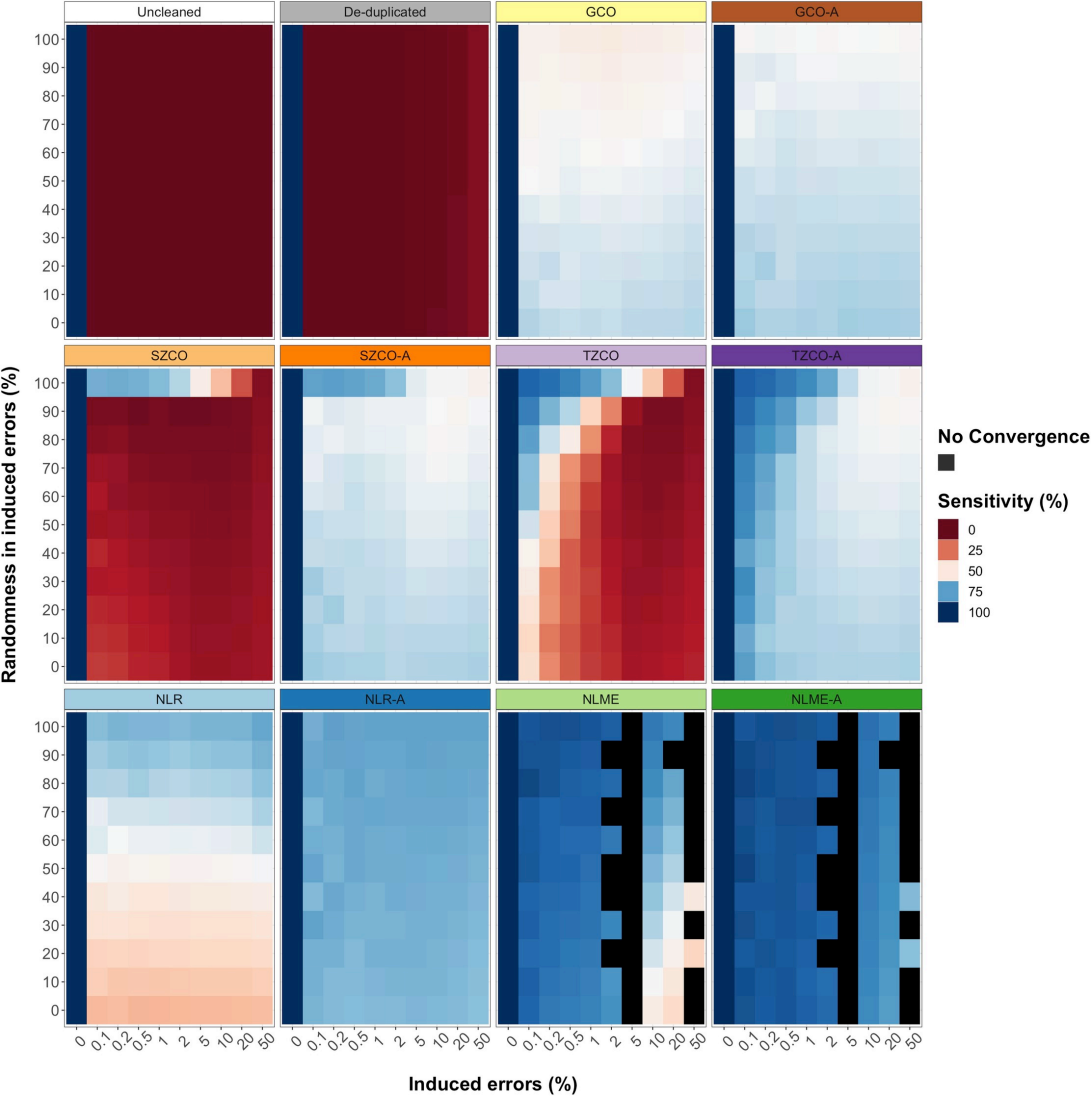
**The sensitivity of uncleaned, de-duplicated data cleaned with five data cleaning approaches with and without our algorithm (A) for longitudinal weight measurements in CLOSER data with different rates and types of simulated errors.** Errors were simulated for 0%, 0.1%, 0.2%, 0.5%, 1%, 2%, 5%, 10%, 20% and 50% of the data. Random errors were simulated between the values of 0.0001 and 500, for 0%, 10%, 20%, 30%, 40%, 50%, 60%, 70%, 80%, 90% and 100% of the overall errors, where fixed errors made up the remaining percentage of errors. Fixed errors comprised of manipulating measurements by multiplying and dividing by 10, 100 and 1000, adding 100 and 1000, converting to the metric and imperial units and transposing the number. Sensitivity calculated as the mean percentage of simulated (true-positive) measurement errors that were correctly identified.

**Fig 4:**
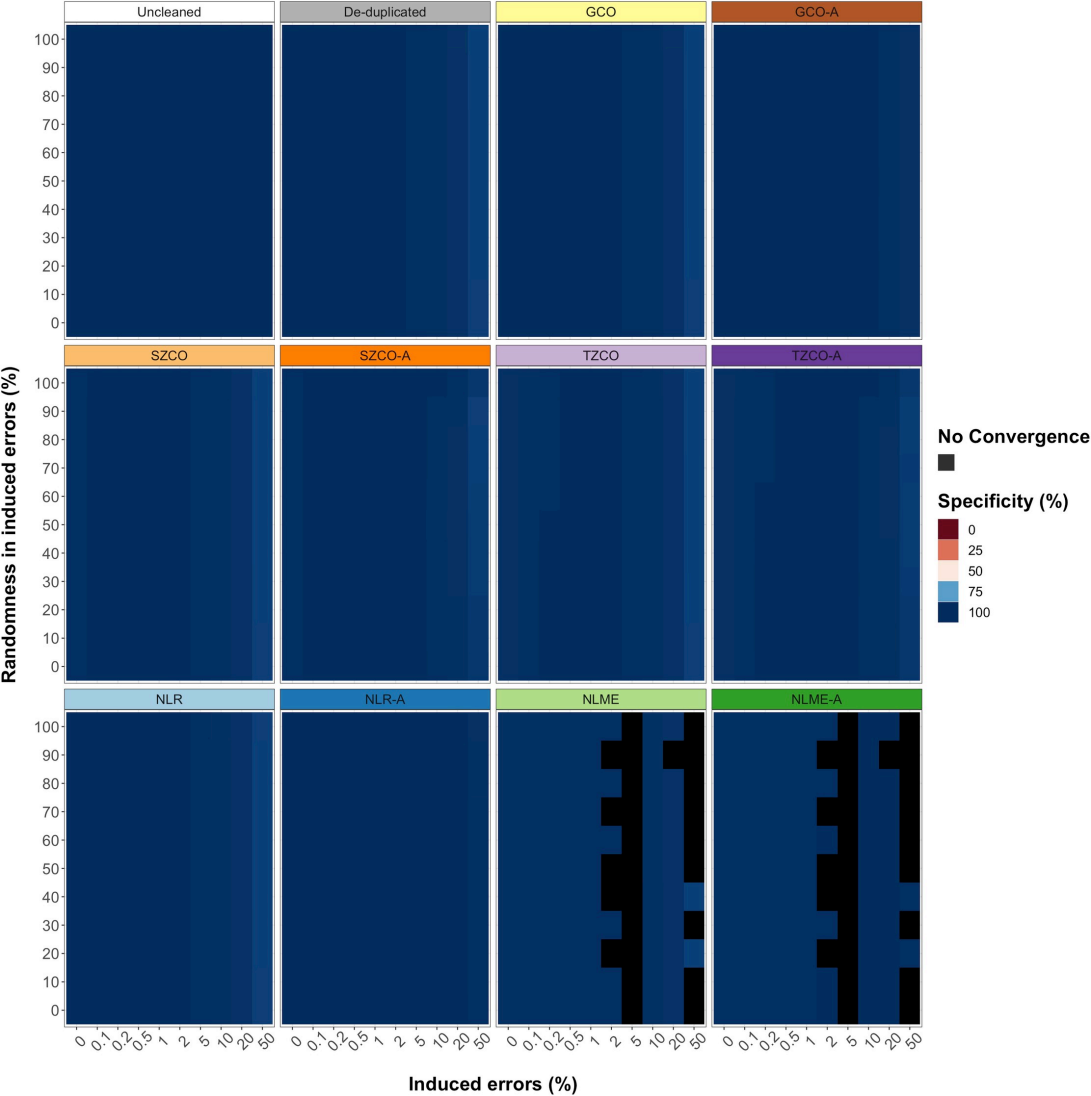
**The specificity of uncleaned, de-duplicated and data cleaned with five data cleaning approaches with and without our algorithm (A) for longitudinal weight measurements in CLOSER data with different rates and types of simulated errors.** Errors were simulated for 0%, 0.1%, 0.2%, 0.5%, 1%, 2%, 5%, 10%, 20% and 50% of the data. Random errors were simulated between the values of 0.0001 and 500, for 0%, 10%, 20%, 30%, 40%, 50%, 60%, 70%, 80%, 90% and 100% of the overall errors, where fixed errors made up the remaining percentage of errors. Fixed errors comprised of manipulating measurements by multiplying and dividing by 10, 100 and 1000, adding 100 and 1000, converting to the metric and imperial units and transposing the number. Specificity was calculated as the mean percentage of non-simulated (true-negative) measurements that were correctly identified.

Table 6, Fig 3 and Fig 4 compare the mean preservation of data, sensitivity and specificity of outlier detection and convergence rate across different rates and types of simulated errors and duplications of the five data cleaning approaches with and without our algorithm in longitudinal growth measurements from CLOSER data. The mean convergence rate across the different rates and types of simulated errors and duplications for all methods was 100% except for the NLME and NLME-A methods, for which it was 76.36%. The SZCO and TZCO methods were the most variable in terms of mean sensitivity across different rates and types of simulated errors and duplications and did not perform well at high error rates and low proportions of randomness. Conversely, the GCO method had a relatively consistent mean sensitivity across all different rates and types of simulated errors and duplications. The mean sensitivity across different rates and types of simulated errors and duplications of the NLME-A method was superior to all other methods when it converged, and when it did not converge the NLME method had the highest mean sensitivity across different rates and types of simulated errors and duplications. The addition of our data cleaning algorithm improved the mean sensitivity across different rates and types of simulated errors and duplications in all methods. Removing duplications alone led to very poor mean sensitivity (0.93%). The overall mean specificity across different rates and types of simulated errors and duplications in all methods was very high (close to 100%) and invariable.

**Table 6:** The mean preservation of data (PD), sensitivity, specificity and convergence rate across different rates and types of simulated errors and duplications of uncleaned, de-duplicated and data cleaned with five data cleaning approaches with and without our algorithm (A) for longitudinal growth measurements from CLOSER data.

Method	Sensitivity (%)	Specificity (%)	PD (%)	Convergence rate (%)
Uncleaned	0.00	100.00	100.00	100.00
De-duplicated	0.93	99.34	92.70	100.00
GCO	57.01	99.34	87.38	100.00
GCO-A	59.85	99.87	92.70	100.00
SZCO	11.02	99.33	92.28	100.00
SZCO-A	60.39	99.57	87.78	100.00
TZCO	25.08	99.29	92.04	100.00
TZCO-A	64.78	99.56	87.93	100.00
NLR	54.03	99.33	87.62	100.00
NLR-A	71.35	99.94	91.47	100.00
NLME	80.03	99.46	88.96	76.36
NLME-A	87.71	99.88	91.76	76.36

Errors were simulated for 0%, 0.1%, 0.2%, 0.5%, 1%, 2%, 5%, 10%, 20% and 50% of the data. Random errors were simulated between the values of 0.0001 and 500, for 0%, 10%, 20%, 30%, 40%, 50%, 60%, 70%, 80%, 90% and 100% of the overall errors, where fixed errors made up the remaining percentage of errors. Fixed errors comprised of manipulating measurements by multiplying and dividing by 10, 100 and 1000, adding 100 and 1000, converting to the metric and imperial units and transposing the number. The preservation of data (PD) describes the percentage of the original data that was preserved. Sensitivity was calculated as the mean percentage of simulated (true-positive) measurement errors that were correctly identified. Specificity was calculated as the mean percentage of non-simulated (true-negative) measurements that were correctly identified. The convergence rate was calculated as the mean percentage of times a method was able to execute correctly.

#### Application of the superior data cleaning method to all datasets

The NLME-A method outperformed other methods in most scenarios, so it was chosen to clean the five datasets. Table 7 presents the percentage of alterations made at each step of the method in each of the datasets. In all datasets, most alterations took place during the first two steps of the algorithm, where identical and similar duplications were removed respectively. The dataset with the most duplicates and errors according to the NLME-A method was the Dogslife heights (16.14% duplicate removals and 3.309% error alterations) while the dataset with the least duplicates was Banfield (0.768% duplicate removals) and the dataset with the least errors was SAVSNET (0.249% error removals). The CLOSER data with simulated duplications and 1% simulated errors after cleaning with the NLME-A method is shown in Fig 1. To visualise all other datasets after cleaning with the NLME-A method, please refer to S1 to S4 Figs.

**Table 7:** The percentage of alterations made to Dogslife, SAVSNET, Banfield and CLOSER data with simulated duplications and 1% simulated errors using the NLME-A data cleaning method.

Step of algorithm	Description of step	Dogslife		SAVSNET	Banfield	CLOSER
		Weights	Heights			
STEP 1	Remove identical duplications	12.52	11.42	10.21	0.671	7.183
STEP 2	Remove similar duplications	1.193	4.716	0.886	0.097	0.119
STEP 3	Replace outliers with the closest correction to the measurement prediction					
Transpose		0.025	0.382	0.000	0.000	0.060
/10		0.108	0.000	0.006	0.011	0.167
/100		0.018	0.000	0.004	0.000	0.132
/1000		0.002	0.000	0.000	0.000	0.041
x10		0.051	0.143	0.008	0.040	0.025
x100		0.000	0.000	0.000	0.000	0.021
x1000		0.000	0.000	0.000	0.000	0.022
-100		0.025	0.004	0.000	0.000	0.016
-1000		0.000	0.000	0.000	0.000	0.038
+100		0.000	0.000	0.000	0.000	0.001
+1000		0.000	0.000	0.000	0.000	0.000
x metric		0.124	1.960	0.032	0.080	0.100
x imperial		0.081	0.200	0.056	0.046	0.045
STEP 4	Remove outliers that jump in size	0.219	0.603	0.142	0.138	0.214
STEP 5	Remove implausible entries	0.005	0.018	0.000	0.017	0.041
Total duplicates removed		13.71	16.14	11.10	0.768	7.301
Total errors removed		0.659	3.309	0.249	0.332	0.924

Simulated errors were made up of 50% random errors and 50% fixed errors. Random errors were simulated between the values of 0.0001 and 500. Fixed errors comprised of manipulating measurements by multiplying and dividing by 10, 100 and 1000, adding 100 and 1000, converting to the metric and imperial units and transposing the number.

## Discussion

Biologically implausible measurements were apparent in all uncleaned growth datasets. The effectiveness of commonly used data cleaning methods varied considerably. By developing and applying a novel, reproducible, adaptable, data cleaning algorithm to established data cleaning methods, these datasets’ errors could be removed or corrected with marked improvements in the sensitivity and specificity of error detection and the preservation of data.

The GCO method benefited from using externally sourced limits to detect outliers and performed consistently across all datasets. The SZCO and TZCO methods relied on z-scores, which are greatly distorted by outlying values distant from the mean and performed worse when the datasets had evidence of more errors or there were larger proportions of simulated errors. The NLR and NLME methods used models built on cleaned subsets of the data and were the most effective out of the non-algorithmic methods across all datasets. The addition of our data cleaning algorithm led to improved data preservation in most datasets, improved the sensitivity and specificity of all methods and was capable of making GS corrections, where simulated errors were returned to their original values.

In general, the NLME-A method was the most sensitive out of all methods but had marginally lower specificity than the NLR-A method. In CLOSER data with simulated errors, the NLME-A method resulted in a mean and standard deviation closer to the original data (without error simulations) than other methods, which demonstrates its ability to detect and modify errors appropriately. The NLME-A method resulted in a greater average percentage of GS error corrections than the NLR-A method, which implies it is superior at making modifications. The evident trade-off between sensitivity and specificity in the NLR-A and NLME-A methods is a common phenomenon and its significance lies within the application of the test that is used; while high sensitivity makes an excellent ‘rule-out’ test, high specificity is a better ‘rule-in’ test. Therefore, if it is vital that minimal false positives are detected, we recommend using the NLR-A method. For all other applications, we recommend using the NLME-A method because it is only marginally less specific but identifies more errors.

Furthermore, the NLME-A method’s general specificity may be higher than is indicated. The CLOSER data was recorded in both metric and imperial units and during certain data collection sweeps, interviewers were able to weigh and/or record the weights of subjects in either kgs or lbs. We propose that the NLME-A method might accurately identify certain instances where the unit of weight had been mis-coded by the interviewer or participant. In support of this, CLOSER data was subject to the application of several different data cleaning protocols before it was made publicly available. CLOSER acknowledge that this led to distortion of the distribution of certain subsets of the data, although attempts were made to rectify these issues [21, 44]. Therefore, it is difficult to understand if the NLME-A method is truly not as specific as the NLR-A method or whether certain errors went undetected by the combination of the CLOSER and specific cohort studies’ data cleaning processes.

The NLME-A method detected duplications, decimal point and unit errors in all datasets but transpose and addition errors seemed to be unique to Dogslife and CLOSER data with simulated errors. The prevalence of errors in our datasets ranged from 0.25% to 3.31%, which is within the same range as previous studies that have identified implausible values in growth data [9–13]. The presence of duplications and errors in medical records emphasises the importance of cleaning datasets even if they have been recorded by professionals. Previous research has reported that duplications can be computer-generated or caused by human error [54] and inaccuracies in weight and height measurements have been attributed to social desirability bias, measurement errors, inaccurate recall and poor measurement equipment [55–62].

The main limitation of the NLME-A method is that the model it was based on failed to converge in 23.64% of CLOSER error simulations. Issues with the convergence of mixed effects models in R are well acknowledged and contrary to statistical premises, are not necessarily an indication that the structure of random effects is over-parameterised [63]. For example, the particular ‘seed’ chosen to randomly simulate errors can affect convergence. We also made no attempt to choose a different weight model for growth in humans than for dogs, although there may be others that could improve convergence. We tested the methods on data with simulated error rates up to 50%, which is likely to be far higher than in real life scenarios. The fact that the NLME model converges in data without error simulations and in CLOSER data with less than 2% simulated errors indicates that the issue is not with the model itself but with the nature of artificial error simulations. A limitation of the algorithm is that it is based on assumptions and require various measurement predictions and limits, implausible measurements, potential error corrections and the limits for transposing numbers to be pre-defined. The assumptions we defined might not be appropriate for other datasets but could be easily modified if necessary. We believe that this algorithm could be adapted not only to different types of growth data but to other forms of temporal data with a functional form.

The NLME-A method allows individual growth trajectories to vary from the population and unlike previously published methods, it does not fail to identify consecutive errors or those within the first or last data entry [11, 12] or require a minimum number of data entries [34]. The method also deals with duplications intelligently by choosing the duplicate that is most likely be correct for that individual. These features offer a reliable and reproducible solution for outlier detection in anthropometric data that has been and continues to be sought after by many researchers [13]. Although we recommend the use of the NLME-A method, we acknowledge that other researchers might not achieve the same sensitivity and specificity in other datasets and that they might need to adapt and improve the models and cut-offs we used for this method. Further work is needed to improve the process of fitting mixed-effects regression models to unclean growth data and to reduce the complexity of ensuring convergence, ideally resulting in the automation of the application of the most appropriate data cleaning method for a given dataset.

This is the first publication that has compared commonly reported data cleaning methods with and without our proposed data cleaning algorithm on data collected with different study designs, from different species, in pre-cleaned data with simulated errors and in uncleaned data with ‘real’ errors. Our methods are easily reproducible and we propose that our algorithm could be adopted in a multitude of different data-related scenarios to improve the stringency of data cleaning.

## Supporting information

S1 File Collection of Dogslife height and weight data. (DOCX)

S2 File Model fitting. (DOCX)

S1 Fig Weights of Labrador Retrievers by age in Dogslife data prior to data cleaning (a) and after data cleaning with the NLME-A method (b).
(TIF)

S2 Fig Heights of Labrador Retrievers by age in Dogslife data prior to data cleaning (a) and after data cleaning with the NLME-A method (b).
(TIF)

S3 Fig Weights of Labrador Retrievers by age in SAVSNET data prior to data cleaning (a) and after data cleaning with the NLME-A method (b).
(TIF)

S4 Fig Weights of Labrador Retrievers by age in Banfield data prior to data cleaning (a) and after data cleaning with the NLME-A method (b).
(TIF)

S1 Table Starting values for the asymptote, lag phase and growth rate of dog and human growth measurements in non-linear regression models for data from Dogslife, SAVSNET, Banfield and CLOSER with and without simulated duplications and 1% simulated errors. Simulated errors were made up of 50% random errors and 50% fixed errors. Random errors were simulated between the values of 0.0001 and 500. Fixed errors comprised of manipulating measurements by multiplying and dividing by 10, 100 and 1000, adding 100 and 1000, converting to the metric and imperial units and transposing the number. Starting values for Dogslife, SAVSNET, Banfield and original CLOSER data were based on a combination of published values and arbitrary guesses. Starting values for CLOSER weights with 1% simulated errors were predicted from non-linear regression models fitted to the original CLOSER data.
(DOCX)

S2 Table Starting values for the asymptote, lag phase and growth rate of dog and human growth measurements in non-linear mixed effects models for data from Dogslife, SAVSNET, Banfield and CLOSER with simulated duplications and 1% simulated errors. Simulated errors were made up of 50% random errors and 50% fixed errors. Random errors were simulated between the values of 0.0001 and 500. Fixed errors comprised of manipulating measurements by multiplying and dividing by 10, 100 and 1000, adding 100 and 1000, converting to the metric and imperial units and transposing the number. Starting values were predicted from non-linear regression models fitted to the data previously.
(DOCX)
