# Supplementary material for: Is it time to stop sweeping data cleaning under the carpet? A novel algorithm for outlier management in growth data
Source: PLoS One. 2020 Jan 24;15(1):e0228154. doi: 10.1371/journal.pone.0228154 (PMC6980495; doi:10.1371/journal.pone.0228154)
Supplement: S1 File — (DOCX) [file pone.0228154.s001.docx]

**Collection of Dogslife height and weight data**

After registration to the study, owners were prompted to complete regular online questionnaires on the Dogslife website about their dog’s morphology, lifestyle and illness incidences. Dog owners were asked to measure their dogs’ weight every time they filled out the questionnaire; monthly for the first 12 months of the dog’s life and every 3 months thereafter. They were given the option to enter the weight of their dog in either kg or lbs in a free text box with a drop-down menu at the side indicating which unit they had chosen. The default unit was kg and owners were not required to answer the question to continue with the questionnaire. If they entered the weight in lbs, it was multiplied by 0.45 and stored in the database as kg. Dog owners were asked to measure their dog’s height to their shoulder every month up to the age of 18 months old and then at 3 years of age. Clear written and pictorial instructions of how to measure the dogs height and weight were given on the website. Owners were given the option to enter the height of their dog in integers of either cm or inches from a drop-down menu and were not allowed to pass the question unless they entered a value. If they entered the height in inches, it was multiplied by 2.54 and stored in the database as cm.
