# Supplementary material for: Is it time to stop sweeping data cleaning under the carpet? A novel algorithm for outlier management in growth data
Source: PLoS One. 2020 Jan 24;15(1):e0228154. doi: 10.1371/journal.pone.0228154 (PMC6980495; doi:10.1371/journal.pone.0228154)
Supplement: S2 File — (DOCX) [file pone.0228154.s002.docx]

## **Model fitting**

**Non-linear regression models**

We used non-linear modified Gompertz regression models for weight data and a non-linear asymptotic regression model for height data in the non-linear regression model cut-off (NLR) and non-linear regression model cut-off with algorithm (NLR-A) data cleaning methods. To ensure model convergence and sensible predicted values, we fitted the models to subsets of each dataset cleaned with the GCO method as described in the main text of the manuscript. Starting values for dog data were estimated by using the rounded means of American Kennel Club breed standards [51] for the asymptote and making arbitrary guesses about the lag phase and growth rate. Starting values for human data were estimated through a two-step improvement process. Original starting values were estimated by using data from the Office for National Statistics [52] for the asymptote and making arbitrary guesses about the lag phase and growth rate. Using these starting values, the model was fitted on the original CLOSER data (without simulated errors) and the parameters from this model were used as starting values for the CLOSER data with simulated errors. See supporting S1 Table for all starting values for non-linear regression models.

Non-linear modified Gompertz regression model for weight:

${W_{i}\left( t \right)= A}_{i}\exp\left( -exp\left[ \frac{\delta_{i}e}{A_{i}} \left\{ \lambda_{i}-t_{i} \right\}+1 \right] \right)$ + ε

$A_{i}$= $\beta_{A0} {+ \beta_{A1}X}_{i}$

$\delta_{i}$= $\beta_{\delta0} {+ \beta_{\delta1}X}_{i}$

$\lambda_{i}$= $\beta_{\lambda0} {+ \beta_{\lambda1}X}_{i}$

ε ~ N(0, σ^2^)

$W_{i}\left( t \right)$= Weight of $i$th dog at time $t$

$A_{i}$= Asymptote (full weight of $i$th dog)

$\delta_{i}$ = Maximum slope (growth rate of $i$th dog)

$\lambda_{i}$ = Lag phase (time period for lag in the growth of the $i$th dog)

$t$ = Age of dog

$\varepsilon$= Residual error

Non-linear asymptotic regression model for height:

$$H_{i}\left( t \right)= A_{i}\left( 1-\exp\left[ -exp\left\{ \delta_{i} \right\}\left\{ t_{i}- \lambda_{i} \right\} \right] \right)+ \varepsilon$$

$A_{i}$= $\beta_{A0} {+ \beta_{A1}X}_{i}$

$\delta_{i}$= $\beta_{\delta0} {+ \beta_{\delta1}X}_{i}$

$\lambda_{i}$= $\beta_{\lambda0} {+ \beta_{\lambda1}X}_{i}$

ε ~ N(0, σ^2^)

$H_{i}\left( t \right)$*=* Height of $i$th dog at time $t$

$A_{i}$= Asymptote (full height of $i$th dog)

$\delta_{i}$ = Logarithm of the rate constant (growth rate of dog)

$\lambda_{i}$ = Lag phase (time period for lag in the growth of the $i$th dog)

$t$ = Age of dog

$\varepsilon$= Residual error

**Non-linear mixed effects models**

We used non-linear modified Gompertz mixed effects models for weight data and a non-linear asymptotic mixed effects model for height data in the non-linear mixed effects model cut-off (NLME) and non-linear mixed effects model cut-off with algorithm (NLME-A) data cleaning methods. To ensure model convergence and sensible predicted values, we fitted the models to subsets of each dataset cleaned with the first two steps of with the NLR-A method as described in the main text of the manuscript and deleted any remaining outliers as defined by the NLR-A method. Starting values for the models were estimated from the parameters given by the non-linear regression models fitted to the data previously. See supporting S2 Table for all starting values for non-linear regression models.

Non-linear modified Gompertz mixed effects model for weight:

${W_{i}\left( t \right)= A}_{i}\exp\left( -exp\left[ \frac{\delta_{i}e}{A_{i}} \left\{ \lambda_{i}-t_{i} \right\}+1 \right] \right)$ + ε

$A_{i}$= $\beta_{A0} {+ \beta_{A1}X}_{i}+u_{\mathrm{Ai}}$

$\delta_{i}$= $\beta_{\delta0} {+ \beta_{\delta1}X}_{i}+u_{\delta i}$

$\lambda_{i}$= $\beta_{\lambda0} {+ \beta_{\lambda1}X}_{i}+u_{\lambda i}$

$u_{\mathrm{Ai}}$ ~ N(0, $\sigma_{A}^{2}$)

$u_{\delta i}$ ~ N(0, $\sigma_{\delta}^{2}$)

$u_{\lambda i}$ ~ N(0, $\sigma_{\lambda}^{2}$)

ε ~ N(0, σ^2^)

$W_{i}\left( t \right)$= Weight of $i$th individual at time $t$

$A_{i}$= Asymptote (full weight of $i$th individual)

$\delta_{i}$ = Maximum slope (growth rate of $i$th individual)

$\lambda_{i}$ = Lag phase (time period for lag in the growth of the $i$th individual)

$t$ = Age of individual

Non-linear asymptotic mixed effects model for height growth:

$$H_{i}\left( t \right)= A_{i}\left( 1-\exp\left[ -exp\left\{ \delta_{i} \right\}\left\{ t_{i}- \lambda_{i} \right\} \right] \right)+ \varepsilon$$

$A_{i}$= $\beta_{A0} {+ \beta_{A1}X}_{i}+u_{\mathrm{Ai}}$

$\delta_{i}$= $\beta_{\delta0} {+ \beta_{\delta1}X}_{i}+u_{\delta i}$

$\lambda_{i}$= $\beta_{\lambda0} {+ \beta_{\lambda1}X}_{i}+u_{\lambda i}$

$u_{\mathrm{Ai}}$ ~ N(0, $\sigma_{A}^{2}$)

$u_{\delta i}$ ~ N(0, $\sigma_{\delta}^{2}$)

$u_{\lambda i}$ ~ N(0, $\sigma_{\lambda}^{2}$)

ε ~ N(0, σ^2^)

$H_{i}\left( t \right)$= Height of $i$th dog at time $t$

$A_{i}$= Asymptote (full height of $i$th individual)

$\delta_{i}$ = Logarithm of the rate constant (growth rate of individual)

$\lambda_{i}$ = Lag phase (time period for lag in the growth of the $i$th individual)

$t$ = Age of individual
