# Supplementary figures and images for: Is it time to stop sweeping data cleaning under the carpet? A novel algorithm for outlier management in growth data
Source: PLoS One. 2020 Jan 24;15(1):e0228154. doi: 10.1371/journal.pone.0228154 (PMC6980495; doi:10.1371/journal.pone.0228154)

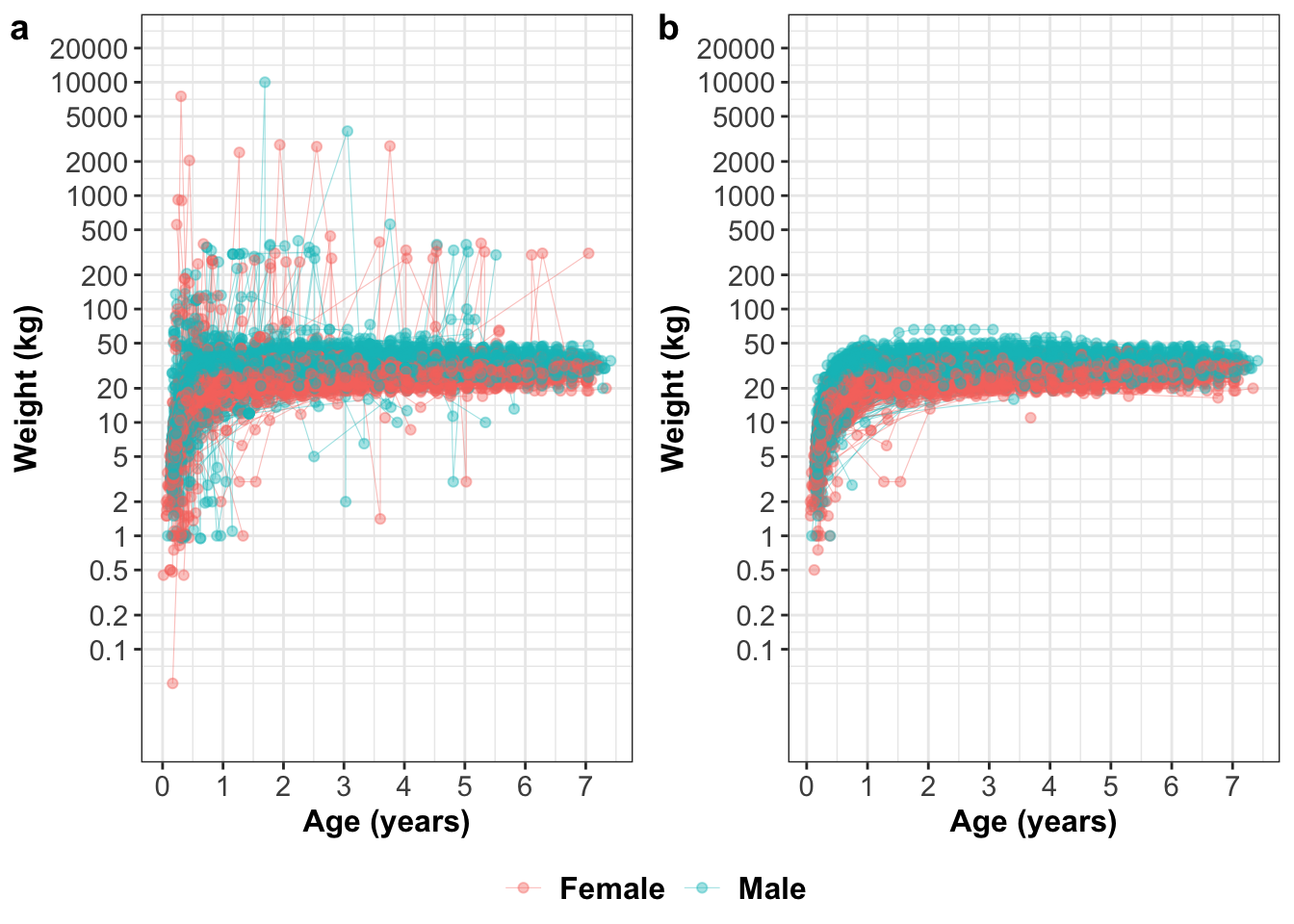

Supplement: S1 Fig — Weights of Labrador Retrievers by age in Dogslife data prior to data cleaning (a) and after data cleaning with the NLME-A method (b). (TIF) [file pone.0228154.s003.tif]

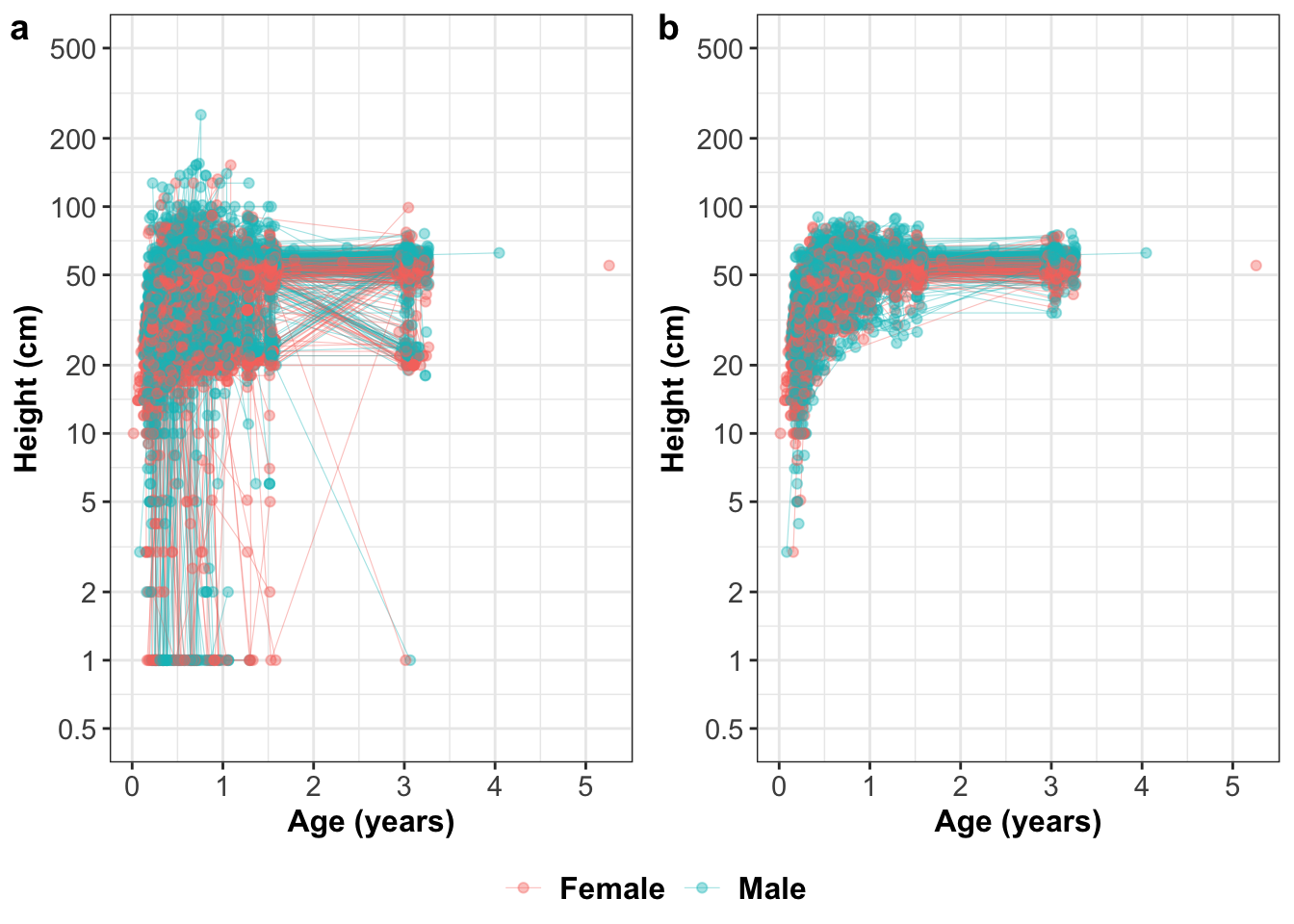

Supplement: S2 Fig — Heights of Labrador Retrievers by age in Dogslife data prior to data cleaning (a) and after data cleaning with the NLME-A method (b). (TIF) [file pone.0228154.s004.tif]

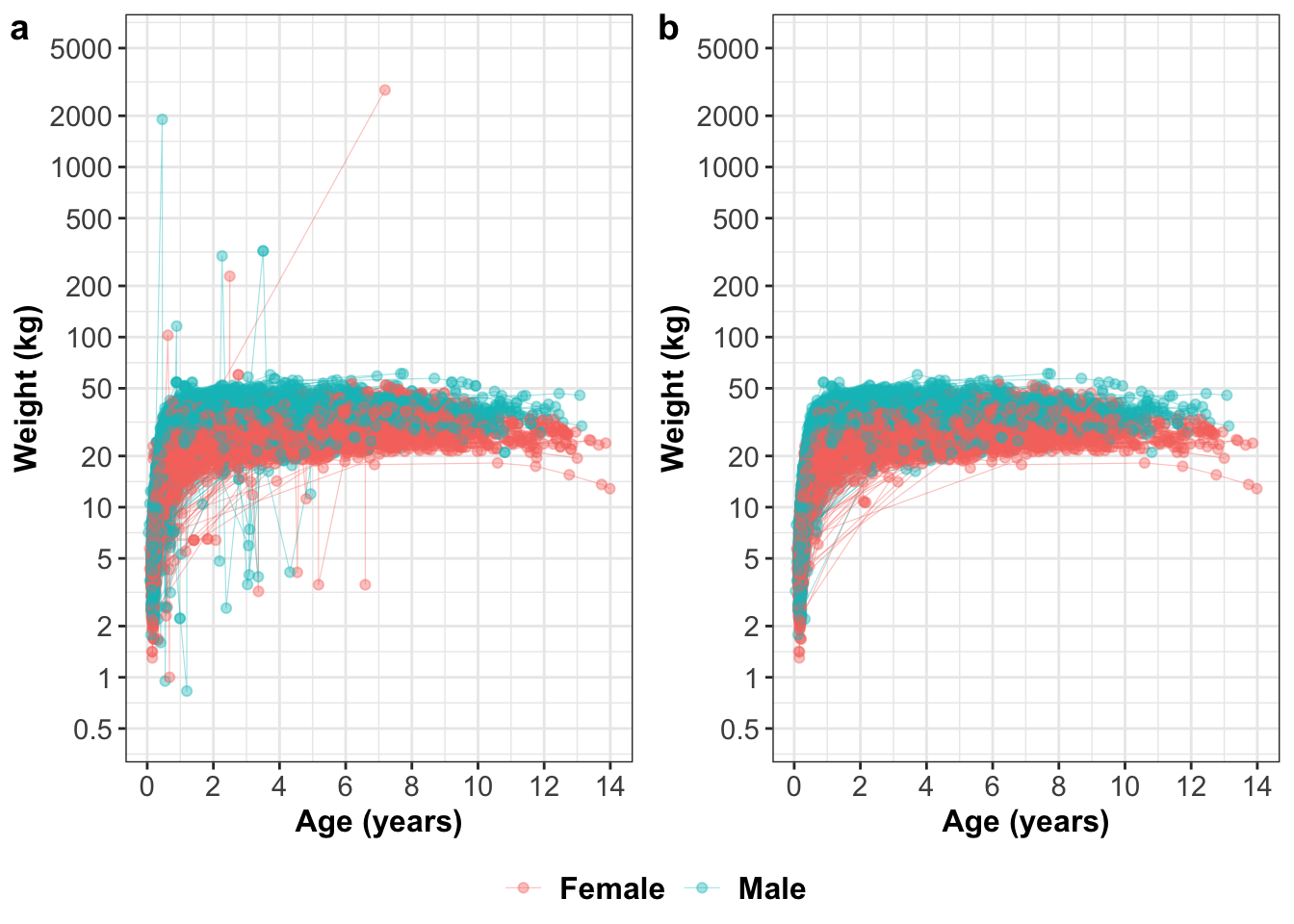

Supplement: S3 Fig — Weights of Labrador Retrievers by age in SAVSNET data prior to data cleaning (a) and after data cleaning with the NLME-A method (b). (TIF) [file pone.0228154.s005.tif]

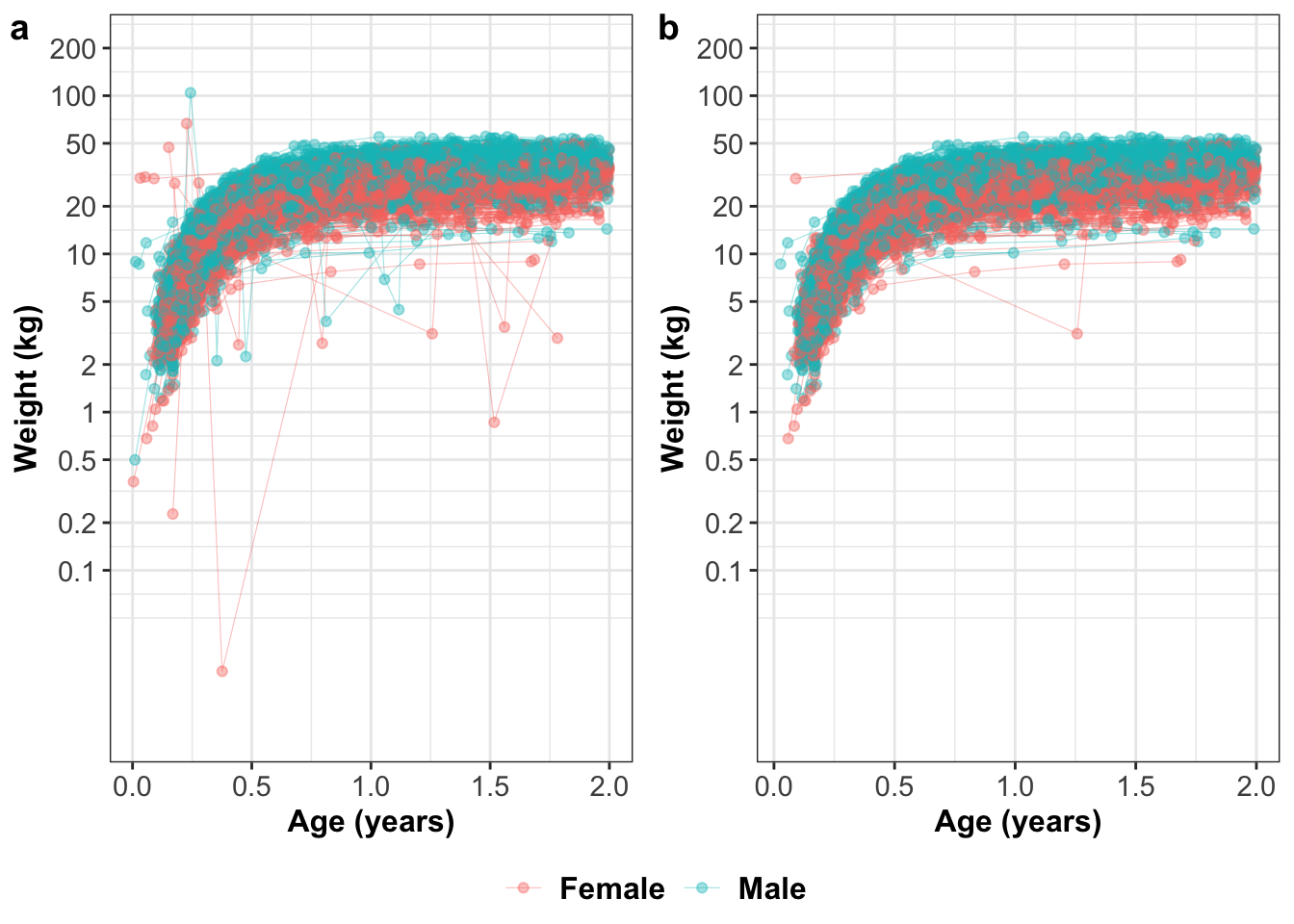

Supplement: S4 Fig — Weights of Labrador Retrievers by age in Banfield data prior to data cleaning (a) and after data cleaning with the NLME-A method (b). (TIF) [file pone.0228154.s006.tif]
