# Supplementary material for: Is it time to stop sweeping data cleaning under the carpet? A novel algorithm for outlier management in growth data
Source: PLoS One. 2020 Jan 24;15(1):e0228154. doi: 10.1371/journal.pone.0228154 (PMC6980495; doi:10.1371/journal.pone.0228154)
Supplement: S1 Table — Simulated errors were made up of 50% random errors and 50% fixed errors. Random errors were simulated between the values of 0.0001 and 500. Fixed errors comprised of manipulating measurements by multiplying and dividing by 10, 100 and 1000, adding 100 and 1000, converting to the metric and imperial units and transposing the number. Starting values for Dogslife, SAVSNET, Banfield and original CLOSER data were based on a combination of published values and arbitrary guesses. Starting values for CLOSER weights with 1% simulated errors were predicted from non-linear regression models fitted to the original CLOSER data. (DOCX) [file pone.0228154.s007.docx]

| Parameter | Dogslife, SAVSNET and Banfield weights | Dogslife heights | Original CLOSER weights | CLOSER weights with 1% simulated errors |
| --- | --- | --- | --- | --- |
| Asymptote: |  |  |  |  |
| Female | 28kg | 57cm | 70.2kg | 69.26kg |
| Male | 33kg | 60cm | 83.6kg | 87.83kg |
| Lag phase: |  |  |  |  |
| Female | 0 days | 0 days | 0 days | -41.77 days |
| Male | 0 days | 0 days | 0 days | 222.10 days |
| Growth rate: |  |  |  |  |
| Female | 0.1 | log(0.02) | 0.1 | 0.010 |
| Male | 0.1 | log(0.02) | 0.1 | 0.010 |
